# The role of wild bees and cavity‐nesting wasps as ecological indicators of the last traditionally managed meadows in Eastern Europe

**DOI:** 10.1002/ece3.70306

**Published:** 2024-10-21

**Authors:** Imre Demeter, Károly Lajos, Adalbert Balog, Miklós Sárospataki

**Affiliations:** ^1^ Lendület Ecosystem Services Research Group, Institute of Ecology and Botany Centre for Ecological Research Vácrátót Hungary; ^2^ Department of Zoology and Ecology, Institute for Wildlife Management and Nature Conservation Hungarian University of Agriculture and Life Science Gödöllő Hungary; ^3^ Department of Horticulture, Faculty of Technical and Human Science Sapientia Hungarian University of Transylvania Tirgu‐Mures Romania

**Keywords:** cavity‐nesting bees, landscape structure, solitary bees, spider prey preference, wild bees

## Abstract

The number of wild bees and cavity‐nesting wasps is abundant in agricultural areas and they contribute significantly to ecosystem services. Due to their specialization in nesting sites and food sources, these groups are sensitive to habitat condition changes and they are therefore important indicators for environmental impact assessments. As semi‐natural habitats are steadily declining and often understudied, their significance for research is increasingly recognized. During this research, the role of wild bee species and cavity‐nesting Hymenopteran taxa as indicators was examined, along the unique combination of high nature value and traditional land use habitats in Eastern Europe, Transylvania. Transects and trap nests were used to test the diversity and abundance of wild bees and cavity‐nesting Hymenopterans to identify possible differences between highly protected and less protected areas. The differences in taxonomic groups between the sites and the potential effects of landscape structure on wild bees and cavity‐nesting Hymenopterans were also assessed. We detected a high diversity of wild bee species and a significant species replacement from one study year to another. Among the nest‐building Hymenopteran taxa, the majority of nests was built by *Trypoxylon* sp. during both study years, with a stronger dominance in the second year. The different taxonomic groups of wild bees and cavity‐nesting Hymenopterans showed differences in their habitat affinities. The majority of the sampled bumblebee species as well as *Trypoxylon* sp. had an affinity towards the study sites located within the highly protected study area. Altogether, we found different habitat preferences for different Hymenopteran groups (both wild bees and wasps) and conclude that these groups definitely have the potential to serve as indicators for differences in the intensity of land use.

## Graphical abstract

The role of wild bees and cavity‐nesting Hymenopteran species as indicators was examined, along the unique combination of high nature value and traditional land use habitats. The different taxonomic groups of wild bees and cavity‐nesting Hymenopterans showed differences in their habitat affinities. Landscape structure had no significant effects on the number of (occupied) brood cells for most of the cavity‐nesting Hymenopteran groups.

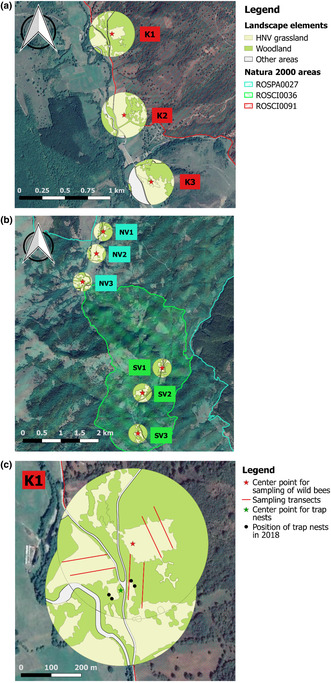

## 1 INTRODUCTION

Insect communities, biomass and species richness decline have been the subject of several studies worldwide in recent years, particularly in developed and developing countries (Forister et al., 2019; Habel et al., 2019; Hallmann et al., 2021; Sánchez‐Bayo & Wyckhuys, 2019). One of the main drivers of this decline is land use change, as agricultural land use has become more intensive, resulting in the decline of high quality semi‐natural habitats (Nieto et al., 2014). Modern agriculture has changed the functioning of agricultural habitat communities by increasing artificial inputs of nutrients and agrochemicals, thereby transforming the biological functions that were originally provided by diverse communities of organisms (Tilman et al., 2001). Agricultural intensification has been successful in that it has helped to meet increasing global food demands by increasing productivity per unit area. On the other hand, negative impacts on the environment and biodiversity have increased significantly (Potts et al., 2010, 2016), many of which relate to ecosystem services (Liu et al., 2018). At the same time, there has been a major transformation of land use in recent decades with the loss of natural habitat elements and a reduction in the complexity of agricultural landscapes (Hoekstra et al., 2005; Tscharntke et al., 1998, 2005).

Semi‐natural grasslands, i.e., grasslands and pastures that are not intensively cultivated and are mainly vegetated by native plant species, are among the richest ecosystems in terms of plant taxa worldwide (Squires et al., 2018; Wilson et al., 2012). The previously mentioned extensively managed grasslands are typical for the Transylvanian region of our study. These areas have been farmed for hundreds of years in a traditional way, which still preserves the biodiversity of the landscape today (Demeter, Balog, Józon, et al., 2021). Land use practices that negatively affect biodiversity, such as small‐plot fields, frequent mowing or extensive fertilization and grazing, are typical of areas close to villages, since the intensively used areas in the immediate vicinity of settlements provide sufficient harvested crops to feed the animals. Choosing the timing of mowing and diversifying it at the habitat level is of paramount importance for habitat conservation, as later mowing favors biodiversity and mimics the traditional timing of mowing (Dahlström et al., 2013; Eriksson et al., 2015; Humbert et al., 2012; Wehn et al., 2018). In areas further away from settlements, traditional, i.e. non‐intensive cultivation is typical, allowing the establishment and maintenance of semi‐natural grasslands. Semi‐natural grasslands are essential habitats for pollinators, as the high diversity of floral resources provide a wide variety of food for pollinator communities throughout the season (Demeter, Balog, & Sárospataki, 2021; Ebeling et al., 2008; Kovács‐Hostyánszki et al., 2016; Nicholls & Altieri, 2012).

In agricultural areas, where landscape structure and land use change frequently, bee diversity and abundance are reduced. As a result, pollination ecosystem function and services are reduced (Hall et al., 2019; Lanner et al., 2020; Potts et al., 2010, 2016). The same applies to changes in the composition of parasitoid or predatory wasp communities, which negatively affect biological control services (Bendixen et al., 2018; Coudrain et al., 2013, 2016; Hoffmann et al., 2020; Stangler et al., 2015). Wasps regulate populations of arthropods, including insect vectors of human diseases, and limit the growth of herbivore populations, which are also essential for a healthy ecosystem (Sumner et al., 2018). Consequently, semi‐natural grassland management is currently considered as one of the key conservation activities for the conservation of biodiversity, pollinators and parasitoid or predatory wasps (Lajos, Samu, et al., 2021; Wehn et al., 2018).

To fill some of the knowledge gaps on pollinator communities in Central and Eastern Europe and as a starting point for conservation efforts, we studied wild bees and cavity‐nesting Hymenopterans in Transylvania (Romania) within and around Natura 2000 protected areas, which were established by the European Union to ensure the long‐term survival of valuable habitats and increase the chances of survival of endangered species (European Commission, 2021). This landscape, which is maintained by traditional land use and cultivation practices, possess a high natural diversity (Hanspach et al., 2014), but at the same time largely understudied. Various but low anthropogenic impacts can alter landscape heterogeneity with negative consequences for pollinator and wasp communities nesting and foraging in their immediate and wider environment. However, these effects may differ between pollinator groups and landscape influences. According to these the following hypotheses were formulated:

1. Are the diversity and abundance of wild bees and cavity‐nesting Hymenopterans higher in the highly protected study area than in the less protected areas?
2. Can the study areas and sites be distinguished based on the occurring taxonomical groups of wild bees and cavity‐nesting Hymenopterans?
3. Is the occurrence of these groups affected by the landscape structure?

## 2 MATERIALS AND METHODS

### 2.1 Study sites

**FIGURE 1:**
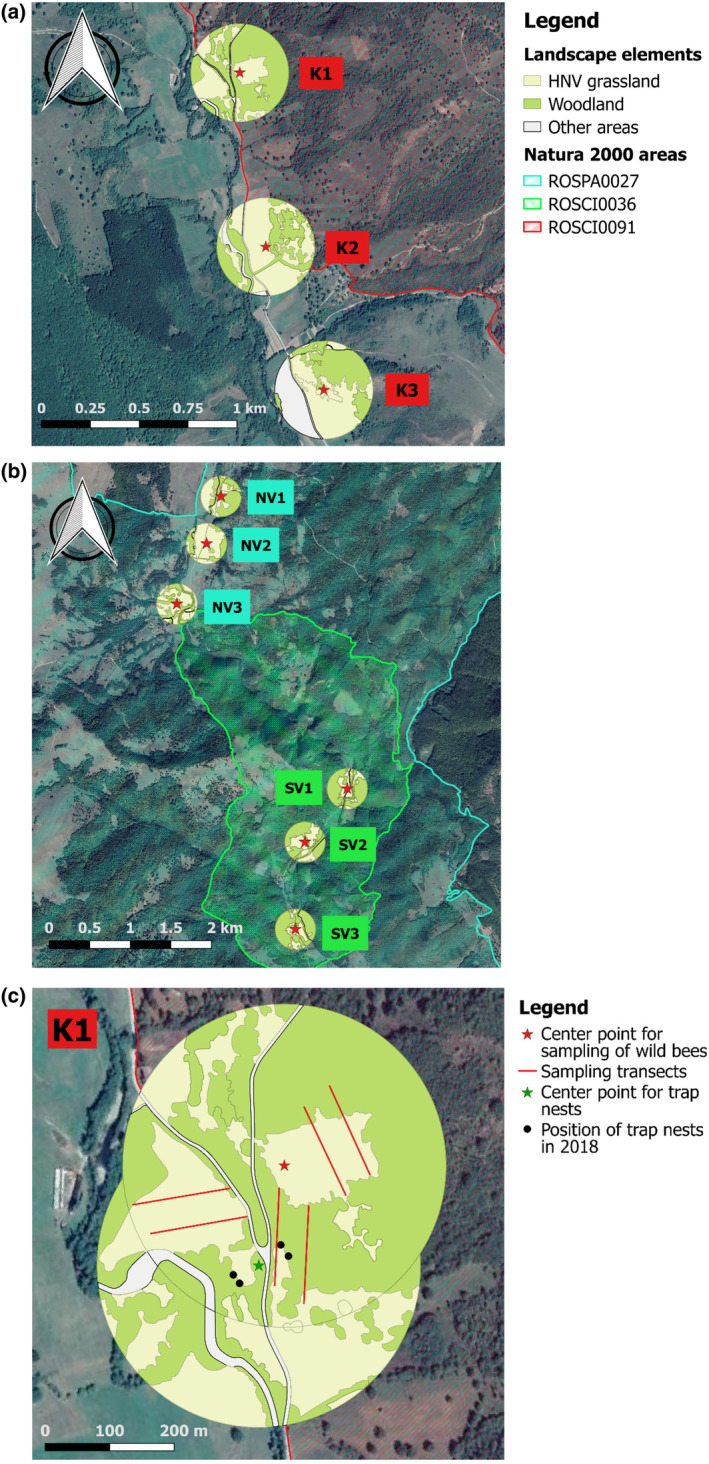
Vector maps of the areas surrounding the nine sampling sites, where the sampling of the wild bees was carried out in 2018 and 2019, located within the three sampling areas (‘K’, ‘NV’ and ‘SV’): (a) the Kormos‐valley (= ‘K’; Natura 2000 site ROSCI0091), and (b) northern Vargyas‐valley (= ‘NV’; Natura 2000 site ROSPA0027), the southern Vargyas‐valley (= ‘SV’; Natura 2000 site ROSCI0036). (c) Approximate arragement of the sampling transects for wild bees in both study years and the position of the trap nests in 2018 at the site K1. Note that the positions of the center points for the vector maps of the trap nests at all sites in both 2018 and 2019 were not at the same positions as for the wild bees.

Our research was carried out in Transylvania (Romania) between 2018 and 2019 in areas with traditional cultivation, extensive farming and high nature value. These areas rich in trees and shrubs have a high plant diversity and density. All study areas were located on the border of the counties of Hargita and Kovászna in a hilly‐mountainous landscape, where the average altitude is 580 m. These areas are mainly used as mowing fields. Mosaic plots rarely exceed one hectare in size and are relatively far from villages. The mosaic plots are mowed at different times of the year, providing a continuous source of food for pollinators throughout the year. The majority of the study sites was located in the Vargyas valley, which is part of the Natura 2000 network. The importance of these high nature value areas has recently increased due to the intensification of agriculture. There were three study areas, which were located within or in close proximity to Natura 2000 sites (Figure 1a,b): The Kormos‐valley (‘K’; Natura 2000 site ROSCI0091), the northern Vargyas‐valley (‘NV’; Natura 2000 site ROSPA0027), and the southern Vargyas‐valley (‘SV’; Natura 2000 site ROSCI0036). The Kormos‐valley was the study area closest to human settlements, with small forest patches and a low number of arable fields in addition to extensive meadows. The study area in the northern Vargyas‐valley was located at a medium distance from human settlements compared to the other two study areas and is mainly used as mowed pastures, grazed in early spring and late autumn. The nature reserve in the southern Vargyas‐valley was located furthest from human settlements and is characterized by forests and mowed areas rich in trees and shrubs.

### 2.2 Sampling of wild bees along a transect

**FIGURE 2:**
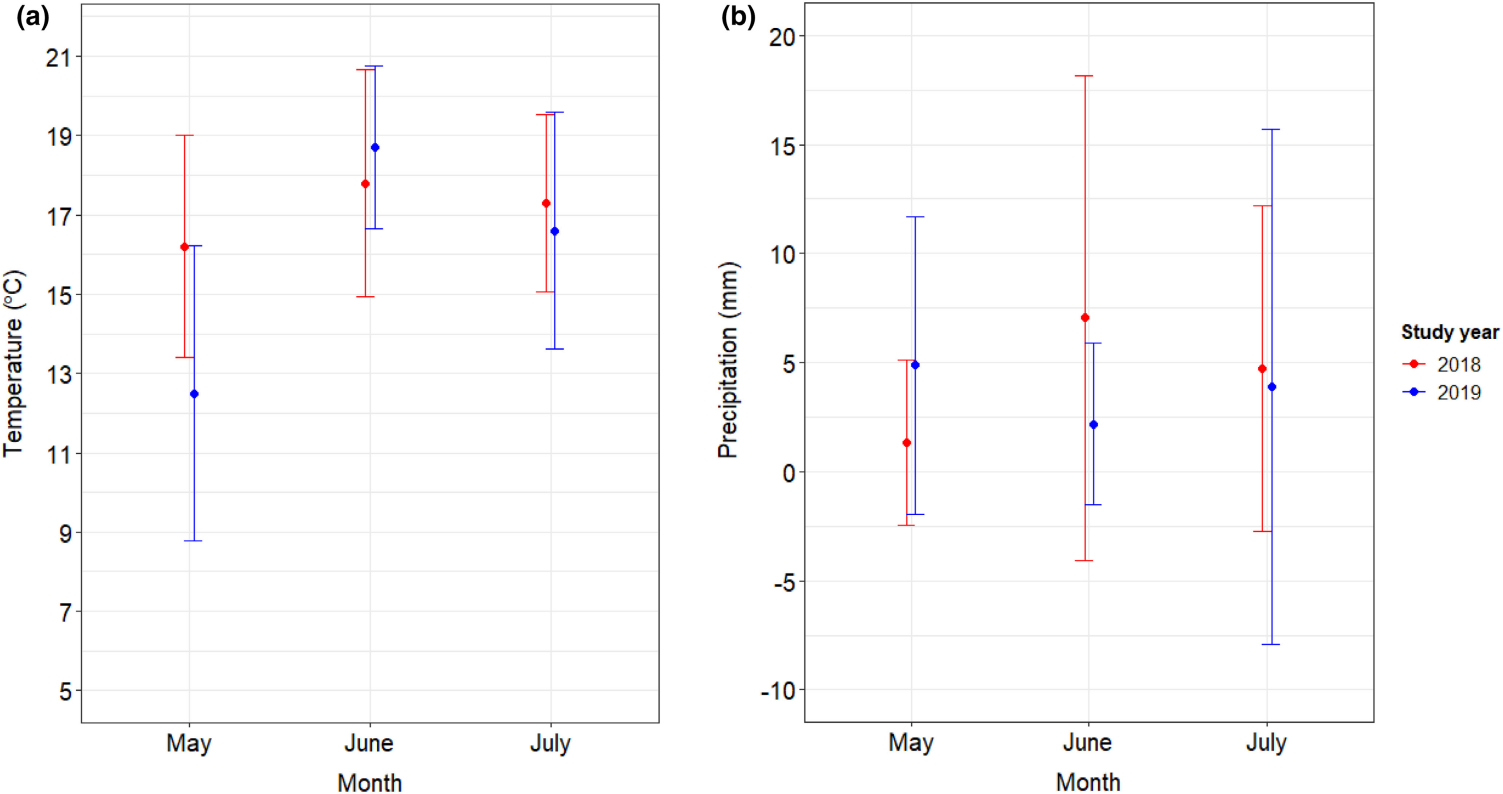
Monthly average (a) temperatures and (b) precipitation measured at Szentegyháza (Vlăhița) during the sampling period in the vicinity of the study area.

The study was conducted over 2 years (2018 and 2019), with sampling carried out once in May, twice in June, and once in July using the transect method. In each study area, three sampling sites were randomly assigned. Three pairs of transects of ca. 200 m in length were assigned at the sampling sites, where two persons sampled simultaneously (Figure 1c). A distance of ca. 50 m was left between the two transects and the transects did not cross. In all cases, sampling took place between 9 and 17 h, in sunny and calm weather, above 13°C and below 32°C. There was a considerable difference in the temperatures measured during May between the 2 years (3.7°C; Figure 2a), while the precipitation was very similar (Figure 2b). During sampling, field‐identifiable individuals (e.g. honeybee, some bumblebee species) were recorded, and those that could not be identified with certainty were placed in 70% alcohol. The wild bees collected were stored in alcohol and sent to taxonomic expert, Zsolt Józan for identification at the end of each year.

### 2.3 Sampling of cavity‐nesting hymenopterans with trap nests

To record the cavity‐nesting Hymenopteran taxa in the three study areas (K, NV and SV), we set up eight study sites in 2018 (the study site NV2 was not sampled in this year) and nine study sites in 2019. Four trap nests in 2018 and three trap nests in 2019 per study site were deployed in 2018 and 2019 (see also Lajos, 2022; Lajos, Demeter, et al., 2021). The trap nests were PVC pipes of ca. 12 cm in diameter and 23 cm in length filled with common reed (*Phragmites australis*), which were installed in May in both years. The reed was cut with a circular saw to a length of 22 cm, ensuring that each reed contained a node. The trap nests were placed 1.5–2 m above ground on nearby shrubs. The trap nests were collected in early September in both years and then stored in a cold shaded outdoor place until January–February. As the weather warmed, the trap nests were taken from this outdoor place and put into a refrigerator at 4–7°C. At the same time, we started processing the trap nests and recording data. We counted the number of reed stems in the trap nests and then cut each reed stem taking care of the nests inside. Where nests were found they were marked with a unique identification code. The following parameters were recorded for each reed: (1) diameter of the reed, (2) number of nest cells, (3) type of nesting material, (4) color of larvae or cocoons, (5) type of food found in the brood, whether nectar‐pollen mixture or dead arthropods (usually spiders). Seven different nest types were identified based on the parameters 3, 4 and 5. In both years, several reeds per nest type were allowed to grow (minimum 2 reeds in 2018, 15–20 reeds in 2019), which were grown at room temperature. Once the individuals were mature, they were placed in 70% ethanol and then sent for identification. The taxonomist Zsolt Józan identified each two genera of Eumenidae (*Ancistrocerus* and *Symmorphus*), Megachilidae (*Megachile* and *Osmia*) and Pomilidae (*Auplopus* and *Dipogon*) as well as one genus of Colletidae (*Hylaeus*) and Crabronidae (*Trypoxylon*). Each nest type was assigned to a genus, except for the two genera *Ancistrocerus* and *Symmorphus* of the subfamily Eumeninae, which could not be distinguished from each other based on the nest parameters.

### 2.4 Landscape structure

The landscape structure surrounding the study sites was quantified by calculating the proportion and edge density, which measures the edge length per area of a landscape element and thus measures the fragmentation of these elements, of woodland patches, the majority of which represents the original natural vegetation in this area. Determining the proportion and edge density of a landscape element is in most cases sufficient to quantify the structure and potential effect of this landscape element on different biological variables or processes (Lajos et al., 2020; Lajos, Demeter, et al., 2021; Lajos, Samu, et al., 2021). To calculate these two landscape metrics, vector maps with a radius of 250 m were created in QGIS 2.18.9 (QGIS Development Team, 2009), around the study sites, using the European Terrestrial Reference System 1989 Lambert Azimuthal Equal Area (ETRS89/ETRS‐LAEA; EPSG: 3035) coordinate reference system (Figure 1a,b). The centers of these circular vector maps were at an approximately equal distance from the sampling transects for the wild bees and from the positions of the trap nests for the cavity‐nesting Hymenopterans (Figure 1c). The vector maps were then transformed into raster maps with a raster cell size of 1 × 1 m, which were used to calculate the landscape metrics ‘Percentage of Landscape’ and ‘Edge Density’ in FRAGSTATS v4.2.1 (McGarigal et al., 2002), applying an eight‐cell neighborhood rule.

### 2.5 Data analyses

All statistical analyses were carried out in R v3.6.3 (R Core Team, 2020). All graphs were created using the R package ‘ggplot2’ (Wickham, 2016). The species diversity of bees was assessed by calculating the Shannon's Diversity Index (SDI) for each site using the R package ‘vegan’ version 2.5–6 (Oksanen et al., 2019). Non‐parametric Wilcoxon signed‐rank tests were used to test for significant differences between the abundances and the SDI of the eight most abundant wild bee taxa, as well as between the abundances of nests built by seven different cavity‐nesting Hymenopteran per site and study year.

Potential habitat affinities (= towards the study areas and sites, respectively) were investigated with principal component analyses (PCAs) and dendrograms using several different functions from the R packages ‘FactoMineR’ (Husson et al., 2014) and ‘factoextra’ (Kassambara & Mundt, 2020). These calculations were based on the numbers of specimens sampled for the eight most abundant wild bee taxa and the numbers of nests built by the seven different cavity‐nesting Hymenopteran taxa.

The relationships between the proportion and edge density of woodland patches around the study sites and the numbers of the eight most abundant wild bee taxa or the (occupied) brood cells of the seven different Hymenopteran nest types were analyzed using generalized linear mixed models (GLMMs) assuming a Poisson distribution from the R package ‘lme4’ (Bates et al., 2015), with the study site and year as random effects. The two landscape metrics of woodland were scaled prior to these GLMMs. The residuals of these GLMMs were tested for uniformity, dispersion and outliers using functions from the R package ‘DHARMa’ (Hartig, 2020). We did not find any significant deviations for the residuals of the tested GLMMs in all cases, where significant relationships were detected.

Finally, we also checked for spatial autocorrelation (Moran's I) using the R package ‘ape’ (Paradis & Schliep, 2019) and reanalyzed the data in all cases, where we found significant relationships. Here, we used Generalized Least Squares (GLS) models by REML from the R package ‘nlme’ (Pinheiro et al., 2013), incorporating a Gaussian correlation structure to correct for spatial autocorrelation. The coordinate reference system used for this analysis was ETRS89/ETRS‐LAEA (EPSG: 3035), the same one as used for mapping.

## 3 RESULTS

### 3.1 Wild bees

**FIGURE 3:**
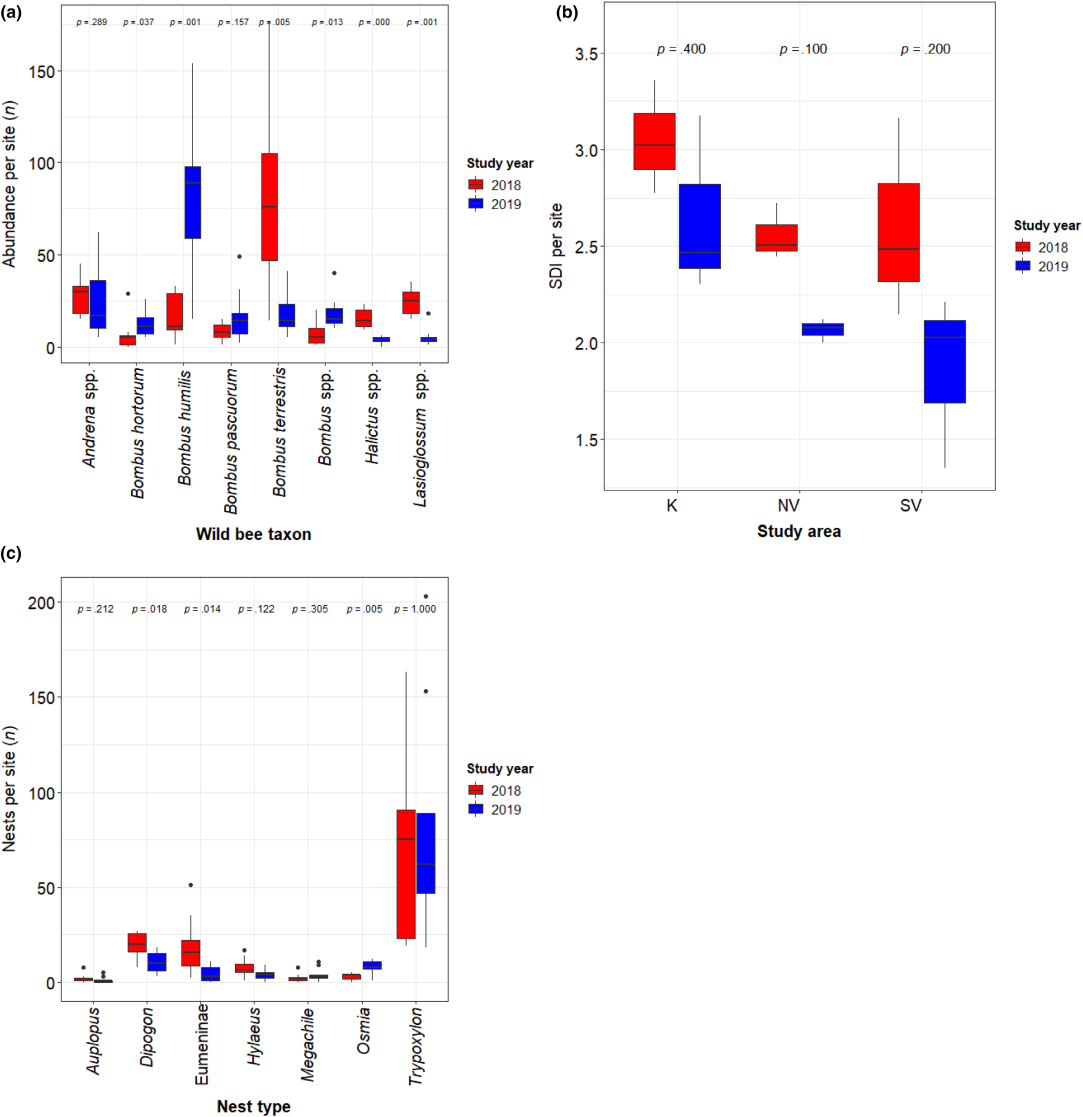
Changes in (a) the abundances per site of the eight most common wild bee taxa, (b) the Shannon Diversity Index (SDI) per site regarding all wild bee species for the three study areas, and (c) the numbers of nests per site and seven different Hymenopteran nest types between the two study years, which were tested for significance using non‐parametric Wilcoxon signed‐rank tests (*p*‐values on top of the figures).

Over the study years 2018 and 2019, we recorded a total number of 3607 individuals and taxonomically identified 157 wild bee species. From these, there were one endangered (EN), 12 near threatened (NT) and 35 data deficient (DD) species (Nieto et al., 2014). In 2018, we sampled 11 bumblebee species with 1046 individuals, while in 2019, 10 bumblebee species with 1335 individuals were detected (there were 12 bumblebee species in total over the two study years).The most common bumblebee species in 2018 was *Bombus terrestris* (Linnaeus, 1758), while *Bombus humilis* (Illiger, 1806) was the most abundant bumblebee species in 2019 (Table 1a). The number of solitary wild bee species was 145, counting 1226 specimens. Among the most common solitary wild bee species were *Andrena flavipes* (Panzer, 1799; *n* = 152), *Andrena ovatula* (Kirby, 1802; *n* = 81), *Eucera longicornis* (Linnaeus, 1758; *n* = 53), *Eucera nigrescens* (Perez, 1879; *n* = 55), *Halictus langobardicus* (Blüthgen, 1944; *n* = 43), *Halictus tumulorum* (Linnaeus, 1758; *n* = 44), *Lasioglossum calceatum* (Scopoli, 1763; *n* = 67) and *Lasioglossum lativentre* (Schenck, 1853; *n* = 37). The eight most abundant wild bee taxa were *Bombus hortorum* (Linnaeus, 1761), *B. humilis*, *Bombus pascuorum* (Scopoli, 1763), *B. terrestris* and the sum of all other species of *Bombus* (= *Bombus* spp.), as well as the sums of all species of *Andrena* spp., *Halictus* spp. and *Lasioglossum* spp. (Table 1a). From these eight groups, the numbers of *B. terrestris*, *Halictus* spp. and *Lasioglossum* spp. dropped significantly from 2018 to 2019, while the abundance of *B. hortorum*, *B. humilis* and other *Bombus* spp. significantly increased (Figure 3a).

**TABLE 1:** Numbers of (a) the most common wild bee taxa, (b) wild bee species at the nine study sites and all sites together, and (c) nest types of cavity‐nesting Hymenopteran taxa during the study years 2018 and 2019.

(a)		
Wild bee taxon	2018	2019
*Andrena* spp.	248	211
*Bombus hortorum*	58	113
*Bombus humilis*	139	741
*Bombus pascuorum*	73	152
*Bombus terrestris*	711	163
Other *Bombus* spp.	65	166
*Halictus* spp.	141	33
*Lasioglossum* spp.	222	49

(b)		
Site	2018	2019
K1	43	26
K2	46	32
K3	43	40
NV1	42	26
NV2	43	26
NV3	42	35
SV1	39	27
SV2	43	27
SV3	58	16
All sites	130	85

(c)		
Nest type	2018	2019
*Auplopus*	18	11
*Dipogon*	158	93
Eumeninae	152	38
*Hylaeus*	61	33
*Megachile*	18	35
*Osmia*	23	73
*Trypoxylon*	560	736

From the 158 taxonomically identified bee species 59 were found in both study years. We recorded 131 species in 2018, from which 72 were unique and not recorded in the following study year, and 86 species in 2019, from which 27 were unique and not recorded in the previous study year (Table 1b). The average number of bee species per site was 45.333 ± 5.431 in 2018 and 29.333 ± 6.764 in 2019. There was a significant decrease in species diversity from 2018 to 2019 (Wilcoxon signed‐rank test: V = 43, *p*‐value = .012) considering all three study areas together, even though this change was not significant looking at the three study areas separately (Figure 3b). In 2018, the numbers of wild bee individuals and species as well as unique species (= only found in the given study area) were the highest in the SV study area. In 2019, however, the number of species and unique species was the highest in the K study area. Although the highest number of individuals was still found in the SV study area. Regarding the species diversity, the highest SDI values were observed at the K study area for both study years (Figure 3b).

### 3.2 Trap nests

We encountered a similar number of Hymenopteran nests (= colonized reeds) in both study years with 990 nests in 2018 and 1019 nests in 2019. The majority of nests were built by *Trypoxylon* sp. in both years (Table 1c; Figure 3c), with a considerably stronger dominance in 2019 (72.23% of all nests) than in 2018 (56.57% of all nests). All specimen reared from the ‘Trypxylon’ nest type were identified as the species *Trypoxylon figulus*, all specimen from the ‘Auplopus’ nest type as *Auplopus carbonarius*, all specimen from the ‘Dipogon’ nest type as *Dipogon bifasciatus* and all specimen from the ‘Megachile’ nest type as *Megachile centuncularis*. We identified two species of *Hylaeus* (*H. arnulatus* and *H. difformis*) and *Osmia* (*O. caerulescens* and *Osmia leaiana*). Specimens reared from the nest type ‘Eumeninae’ were identified as three species of each *Ancistrocerus* (*A. antilope*, *A. nigricornis* and *A. oviventris*) and *Symmorphus* (*S. crassicornis*, *S. debilitatus* and *S. fuscipes*). The numbers of nests built by *Dipogon* sp. and Eumeninae (potter wasps) were all significantly lower in 2019 than in 2018, whereas the numbers of nests built by the solitary bee taxon *Osmia* sp. was significantly higher in 2019 than in 2018 (Table 1c; Figure 3c).

### 3.3 Habitat affinities

The PCAs showed that the majority of the sampled bumblebee species had an affinity towards the study sites located within the highly protected area south of the Vargyas‐gorge (study area SV with the study sites SV1–3 within the Natura 2000 area ROSCI0036) in both study years (Figure S1a,c). In contrast to this, the three genera of more common solitary bees (*Andrena* spp., *Halictus* spp. and *Lasioglossum* spp.) mostly showed stronger affinities towards the less protected and thus more human‐influenced study sites (study areas K and NV with the study sites K1–3 and NV1–3) and were usually less common at the sites located within the highly protected area in both study years (Figure S1a,c). The clustering of the study sites was clearer in 2018 than it was in 2019 (Figure S1b,d).

From the cavity‐nesting Hymenopteran taxa, only nests of the wasp *Trypoxylon* sp. showed affinity towards the study sites located within the highly protected area (= SV1–3), which had the highest nest‐numbers mostly at these study sites, especially in 2019 (Figure S2a,c). The majority of the other cavity‐nesting Hymenopteran taxa, however, was more strongly associated with those study sites, where the disturbance by humans was stronger (= K1–3 and NV1–3; Figure S2a,c). In contrast to the wild bees, the clustering of the study sites was clearer in 2019 than it was in 2018 (Figure S2b,d).

**FIGURE 4:**
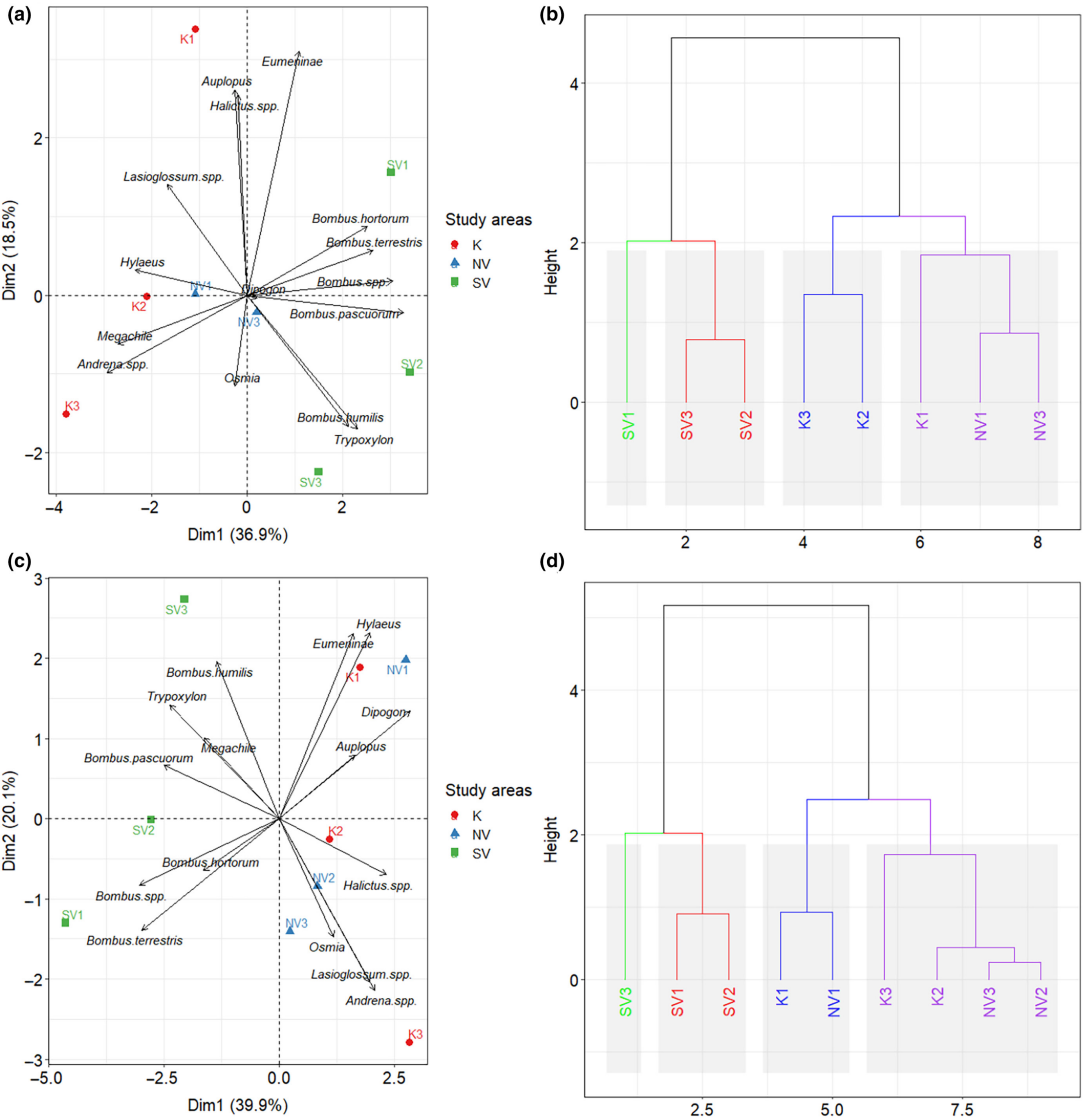
Affinities of both the eight most abundant wild bee and seven different Hymenopteran nest types towards the study sites and areas in 2018 (a, b) and 2019 (c, d). The affinities were determined using principal component analyses (PCAs) and dendrograms. The colors in the dendrograms do not correspond with the colors of the study areas in the figures depicting the PCAs.

Combining the datasets of wild bees and cavity‐nesting Hymenopterans resulted in a much clearer clustering and thus better distinction of the study sites SV1–3 from the other, more human‐influenced study sites (K1–3 and NV1–3) in both the PCAs (Figure 4a,c) and the dendrograms (Figure 4b,d).

### 3.4 Sensitivity to landscape structure

The majority of both wild bees and cavity‐nesting Hymenopterans were not significantly affected by the landscape structure surrounding the study sites. When looking at the data from both study years together, only four taxonomic groups were significantly affected by the proportion or edge density of woodland patches around the study sites: The numbers of *B. pascuorum* and *Lasioglossum* spp. in the case of the wild bees (Table 2), and the brood cell numbers of nests built by potter wasps (Eumeninae) and the predatory wasp *Trypoxylon* sp. (Table 3). From these groups only the potter wasps had a significant positive correlation with the edge density, while all other three groups showed significant relationships with the proportion of woodland patches. These latter relationships were mostly positive, with the exception of *Lasioglossum* spp., where it was negative.

**TABLE 2:** Relationships between the proportion and edge density of woodland patches, located within a 250 m radius around the study sites, and the numbers of the eight most abundant wild bee taxa in both study years (2018 and 2019).

Wild bee taxon	Predictors	Estimate	SE	*z*‐value	*p*‐value
*Andrena* spp.	*Intercept*	3.166	0.114	27.682	**<.001**
	Proportion	−0.013	0.006	−1.954	.051
	Edge density	−0.001	0.001	−0.966	.334
*Bombus hortorum*	*Intercept*	2.012	0.318	6.319	**<.001**
	Proportion	0.018	0.013	1.427	.153
	Edge density	−0.002	0.003	−0.587	.557
*Bombus humilis*	*Intercept*	3.423	0.636	5.386	**<.001**
	Proportion	0.011	0.012	0.943	.346
	Edge density	0.003	0.003	1.122	.262
*Bombus pascuorum*	*Intercept*	2.222	0.283	7.853	**<.001**
	Proportion	0.043	0.007	5.795	**<.001**
	Edge density	−0.002	0.002	−1.258	.208
*Bombus terrestris*	*Intercept*	3.460	0.575	6.017	**<.001**
	Proportion	0.014	0.013	1.110	.267
	Edge density	0.001	0.003	0.253	.800
*Bombus* spp.	*Intercept*	2.329	0.384	6.062	**<.001**
	Proportion	0.009	0.011	0.849	.396
	Edge density	−0.001	0.002	−0.224	.823
*Halictus* spp.	*Intercept*	2.009	0.523	3.842	**<.001**
	Proportion	0.005	0.006	0.751	.453
	Edge density	0.001	0.001	0.641	.522
*Lasioglossum* spp.	*Intercept*	2.427	0.537	4.520	**<.001**
	Proportion	−0.014	0.004	−3.680	**<.001**
	Edge density	0.000	0.001	−0.049	.961

*Note*: These relationships were tested using generalized linear mixed models (GLMMs) assuming a Poisson distribution and the study site and year as random effects. Significant *p*‐values are marked bold.

**TABLE 3:** Relationships between the proportion and edge density of woodland patches, located within a 250 m radius around the study sites, and the numbers of the occupied brood cells of the seven Hymenopteran nest types in both study years (2018 and 2019).

Nest type	Predictors	Estimate	SE	*z*‐value	*p*alue
*Auplopus*	*Intercept*	1.517	0.569	2.668	**.008**
	Proportion	−0.005	0.039	−0.133	.894
	Edge density	0.001	0.008	0.151	.880
*Dipogon*	*Intercept*	3.771	0.149	25.357	**<.001**
	Proportion	0.002	0.006	0.309	.758
	Edge density	0.000	0.001	0.119	.905
Eumeninae	*Intercept*	3.140	0.524	5.989	**<.001**
	Proportion	0.024	0.017	1.416	.157
	Edge density	0.009	0.004	2.440	**.015**
*Hylaeus*	*Intercept*	3.026	0.206	14.711	**<.001**
	Proportion	0.019	0.017	1.150	.250
	Edge density	0.005	0.003	1.448	.148
*Megachile*	*Intercept*	2.279	0.444	5.133	**<.001**
	Proportion	0.016	0.019	0.853	.394
	Edge density	−0.001	0.004	−0.235	.814
*Osmia*	*Intercept*	3.372	0.407	8.279	**<.001**
	Proportion	−0.009	0.008	−1.223	.221
	Edge density	0.000	0.002	−0.148	.883
*Trypoxylon*	*Intercept*	5.539	0.221	25.072	**<.001**
	Proportion	0.030	0.014	2.191	**.029**
	Edge density	0.000	0.003	0.120	.905

*Note*: These relationships were tested using generalized linear mixed models (GLMMs) assuming a Poisson distribution, with the study site and year as random effects. Significant *p*‐values are marked bold.

We detected significant spatial autocorrelations for *B. pascuorum* in 2018 (*p*‐value = .004), *Lasioglossum* spp. in both study years (*p*‐value for 2018 = .016; *p*‐value for 2019 = .036) and the *Trypoxylon* nest type in 2019 (*p*‐value = .021). The GLS models corrected for these spatial autocorrelations also showed a significant positive relationship between the proportion of woodland and the numbers of *B. pascuorum* (Estimate = 0.156; SE = 0.048; *t*‐value = 3.226; *p*‐value = .015) and a significant negative correlation for the numbers of *Lasioglossum* spp. in 2018 (Estimate = −0.112; SE = 0.036; *t*‐value = −3.109; *p*‐value = .017). However, the GLS models showed non‐significant correlations for *Lasioglossum* spp. in 2019 (Estimate = −0.156; SE = 0.092; *t*‐value = −1.691; *p*‐value = .135) as well as for *Trypoxylon* in 2019 (Estimate = −5.321; SE = 5.508; *t*‐value = −0.966; *p*‐value = .366). But, the GLS using REML without a Gaussian correlation structure provided a better fit and showed a significant positive relationship for *Trypoxylon* (Estimate = 12.682; SE = 5.216; *t*‐value = 2.431; *p*‐value = .045).

## 4 DISCUSSION

### 4.1 Wild bees and cavity‐nesting hymenopterans

We encountered considerable differences in the species diversity of wild bees between the two study years, which was associated with the disappearance of certain species and the occurrence of other ones. This change can be explained in different ways. One factor can be the food resource availability during larval development, offspring that fail to accumulate sufficient adipose tissue are more likely to die during development (Bosch, 2008) and overwintering, and smaller individuals have more difficulty finding and protecting nest sites (Bosch & Kemp, 2002; Bosch & Vicens, 2006). Another factor might be the differences in climate between the two study years. Changes in climate can significantly affect the abundance of wild bees (Papanikolaou et al., 2017). Specifically, increases in temperature lead to a decrease in bee diversity, even when corrected for the effects of phenology, confirming worrying reports of potential negative effects of climate warming on wild bees (Potts et al., 2010). Similar declines have been observed in studies investigating the effects of climate change on wild bees using long‐term data (Bartomeus et al., 2013). In our case, *B. terrestris*, *Halictus* spp. and *Lasioglossum* spp. and the total number of wild bee species showed significant declines in 2019, while the abundance of e.g. *B. humilis* strongly increased. Temperature might have a direct effect on bee development, survival, distribution and abundance (Bale et al., 2002). Climate warming is expected to affect activity patterns of different species differently (Rader et al., 2013). The size and connectivity of forest habitat patches are also important for pollinator survival in drought years (Pan et al., 2013), as loss of forest cover in warmer regions accelerates species movement to higher elevations (Guo et al., 2018). Although regions dominated by higher proportions of forests may to some extent dampen the effects of climate warming (Ganuza et al., 2022).

We found a similar number of Hymenopteran nests in both study years, but with differences in the proportion of the different nest types. The majority of nests were built by *Trypoxylon* sp. in both years, with a considerably stronger dominance in 2019. Assessments from a similarly heterogeneous landscape, dominated by grasslands and forests (Schleswig‐Holstein, northern Germany), reported the digger wasp *T. figulus*, which was also the species found at our study sites, as the most abundant (Kruess & Tscharntke, 2002). Our results are also consistent with the results reported in a study from central‐western Spain (Tormos et al., 2005), where *Trypoxylon* was the most abundant genus (272 nests, 72.9%). In another study conducted in central Germany in a region with 44% of the area under agricultural management, *Trypoxylon* was also the most abundant spider‐hunting genus (Hoffmann et al., 2020). However, two other studies conducted in high‐intensity agricultural landscapes situated in southwestern Germany reported that the majority of nests of cavity‐nesting hymenopterans was built by the solitary bees *Megachile* and *Osmia*, which were relatively rare in our case, while the genus *Trypoxylon* was only the third most abundant (Gathmann et al., 1994; Steffan‐Dewenter, 2002). Altogether, and according to our first hypothesis, our results highlight that higher diversity of wild bees and cavity‐nesting Hymenopterans can be detected in these protected areas – low or no disturbance increasing diversity – but also predicts that the pollinator communities might be highly sensitive to short term change in climate that might occur from 1 year to another, some species however are less sensitive to these rapid changes.

### 4.2 The occurrence of different taxonomic groups between sites

According to our findings, it can be concluded that the study areas and sites can be distinguished based on the occurrence of different taxonomical groups of wild bees and cavity‐nesting Hymenopterans, which confirmed our second hypothesis The majority of the sampled bumblebee species had an affinity towards the study sites located within the highly protected area south of the Vargyas gorge (= study sites SV1–3 within the Natura 2000 area ROSCI0036). In contrast to this, the three genera of more common solitary bees (= *Andrena*, *Halictus* and *Lasioglossum* spp.) mostly showed stronger affinities towards the less protected and thus more human‐influenced study areas (= study sites K1–3 and NV1–3) and were usually less common at the sites located within the highly protected area. It can also be stated that *Trypoxylon* sp. showed a higher affinity towards the highly protected area (especially in 2019), while the other cavity‐nesting Hymenopteran taxa mostly showed higher affinities towards the less protected study areas.

### 4.3 Habitat affinities and sensitivities to landscape structure

For the wild bees, only the numbers of *B. pascuorum* and *Lasioglossum* spp. were significantly affected by the proportion of woodland patches in both study years. A positive relationship was found for *B. pascuorum*, while the effect for *Lasioglossum* spp. was negative. Among the cavity‐nesting Hymenoptera taxa, the edge density of woodland patches had a significantly positive effect on the number of brood cells built by potter wasps (Eumeninae) and the proportion of woodland patches had a significantly positive effect on the number of brood cells built by the wasp Trypoxylon sp., while the other cavity‐nesting Hymenopteran taxa were unaffected by the landscape structure. Similarly, the study of Fabian et al. (2013) reported that forest cover had a positive effect on the species richness of wasps. Schüepp et al. (2011) also found that in areas with a high proportion of woody habitats, wasp abundance more than doubled and diversity more than tripled compared to areas with low proportions of woody habitats. Furthermore, two other studies reported that Eumenid, Pompilid and Sphecid wasp abundance was high in forest edges providing natural nesting habitat, but low in grass strips where suitable nesting habitat was scarce (Holzschuh et al., 2009, 2010). Altogether, we can conclude that in our case the majority of the wild bee and cavity‐nesting Hymenopteran taxa were insensitive to landscape structure, which indicates that the effect of the quality of the studied habitats seems to be more important than their structure.

## 5 CONCLUSIONS

Altogether, our 2‐year study gave a first insight in the habitat preferences of different Hymenopteran groups. However, in future studies several other factors have to be tested to see the whole picture in these preferences. Probably, factors as rapid changes in climate, human influence and competition with other groups (i.e. honeybees) might have significant effects even in short time periods (from 1 year to another, as in our case). We can conclude that several aspects and the role of wild bees as well as cavity‐nesting Hymenopteran taxa as indicators in semi‐natural habitats were observed in this traditionally used heterogeneous landscape. As only few such habitats still remain in Europe and as the maintenance of insect biodiversity is crucial for well‐functioning ecosystems, our results can be important for future researches in areas both less or more strongly influenced by humans.

## AUTHOR CONTRIBUTIONS

**Imre Demeter:** Conceptualization (equal); data curation (equal); formal analysis (equal); funding acquisition (equal); investigation (equal); methodology (equal); project administration (equal); resources (equal); software (equal); supervision (equal); validation (equal); visualization (equal); writing – original draft (equal); writing – review and editing (equal). **Károly Lajos:** Conceptualization (equal); data curation (equal); investigation (equal); methodology (equal); resources (equal); software (equal); validation (equal); visualization (equal); writing – original draft (equal); writing – review and editing (equal). **Adalbert Balog:** Conceptualization (equal); data curation (equal); formal analysis (equal); funding acquisition (equal); investigation (equal); methodology (equal); project administration (equal); resources (equal); software (equal); supervision (equal); validation (equal); visualization (equal); writing – original draft (equal); writing – review and editing (equal). **Miklós Sárospataki:** Conceptualization (equal); data curation (equal); formal analysis (equal); funding acquisition (equal); investigation (equal); methodology (equal); project administration (equal); resources (equal); supervision (equal); visualization (equal); writing – original draft (equal); writing – review and editing (equal).

## CONFLICT OF INTEREST STATEMENT

The authors declare that they have no known competing financial interests or personal relationships that could have appeared to influence the work reported in this paper.

## Supporting information


Figures S1–S2

## Data Availability

The datasets are available at Drayad: https://datadryad.org/stash/share/El3Bw0bUh3kn_3r0areLkPXHj0QJimUTVfH1XVczpwY.

## References

[ece370306-bib-0001] Bale, J. S. , Masters, G. J. , Hodkinson, I. D. , Awmack, C. , Bezemer, T. M. , Brown, V. K. , Butterfield, J. , Buse, A. , Coulson, J. C. , Farrar, J. , Good, J. E. G. , Harrington, R. , Hartley, S. , Jones, T. H. , Lindroth, R. L. , Press, M. C. , Symrnioudis, I. , Watt, A. D. , & Whittaker, J. B. (2002). Herbivory in global climate change research: Direct effects of rising temperature on insect herbivores. Global Change Biology, 8, 1–16. 10.1046/j.1365-2486.2002.00451.x

[ece370306-bib-0002] Bartomeus, I. , Park, M. G. , Gibbs, J. , Danforth, B. N. , Lakso, A. N. , & Winfree, R. (2013). Biodiversity ensures plant–pollinator phenological synchrony against climate change. Ecology Letters, 16, 1331–1338. 10.1111/ele.12170 23968538

[ece370306-bib-0003] Bates, D. , Mächler, M. , Bolker, B. , & Walker, S. (2015). Fitting linear mixed‐effects models using lme4. Journal of Statistical Software, 67, 1–48. 10.18637/jss.v067.i01

[ece370306-bib-0004] Bendixen, U. , Jauker, F. , Lanzen, J. , Warzecha, D. , Wolters, V. , & Diekötter, T. (2018). Prey‐dependent benefits of sown wildflower strips on solitary wasps in agroecosystems. Insect Conservation and Diversity, 11, 42–49. 10.1111/icad.12270

[ece370306-bib-0005] Bosch, J. (2008). Production of undersized offspring in a solitary bee. Animal Behaviour, 75, 809–816. 10.1016/j.anbehav.2007.06.018

[ece370306-bib-0006] Bosch, J. , & Kemp, W. (2002). Developing and establishing bee, species as crop pollinators: The example of *Osmia* spp. (Hymenoptera: Megachilidae) and fruit trees. Bulletin of Entomological Research, 92, 3–16. 10.1079/BER2001139 12020357

[ece370306-bib-0007] Bosch, J. , & Vicens, N. (2006). Relationship between body size, provisioning rate, longevity and reproductive success in females of the solitary bee *Osmia cornuta* . Behavioral Ecology and Sociobiology, 60, 26–33. 10.1007/s00265-005-0134-4

[ece370306-bib-0008] Coudrain, V. , Herzog, F. , & Entling, M. (2013). Effects of habitat fragmentation on abundance, larval food and parasitism of a spider‐hunting wasp. PLoS One, 8, e59286. 10.1371/journal.pone.0059286 23516622 PMC3597609

[ece370306-bib-0009] Coudrain, V. , Rittiner, S. , Herzog, F. , Tinner, W. , & Entling, M. H. (2016). Landscape distribution of food and nesting sites affect larval diet and nest size, but not abundance of *Osmia bicornis* . Insect Science, 23, 746–753. 10.1111/1744-7917.12238 25973721

[ece370306-bib-0010] Dahlström, A. , Iuga, A.‐M. , & Lennartsson, T. (2013). Managing biodiversity rich hay meadows in the EU: A comparison of Swedish and Romanian grasslands. Environmental Conservation, 40, 194–205. 10.1017/S0376892912000458

[ece370306-bib-0012] Demeter, I. , Balog, A. , & Sárospataki, M. (2021). Variation of small and large wild bee communities under honeybee pressure in highly diverse natural habitats. Frontiers in Ecology and Evolution, 9, 750236. 10.3389/fevo.2021.750236

[ece370306-bib-0011] Demeter, I. , Balog, A. , Józon, Z. , & Sárospataki, M. (2021). Comparison of wild bee communities of three semi‐natural meadow habitats at Harghita‐Covasna region, Transylvania, Romania. Acta Zoologica Academiae Scientiarum Hungaricae, 67(2), 161–175. 10.17109/AZH.67.2.161.2021

[ece370306-bib-0013] Ebeling, A. , Klein, A. , Schumacher, J. , Weisser, W. , & Tscharntke, T. (2008). How does plant richness affect pollinator richness and temporal stability of flower visits? Oikos, 117, 1808–1815. 10.1111/j.1600-0706.2008.16819.x

[ece370306-bib-0014] Eriksson, O. , Bolmgren, K. , Westin, A. , & Lennartsson, T. (2015). Historic hay cutting dates from Sweden 1873–1951 and their implications for conservation management of species‐rich meadows. Biological Conservation, 184, 100–107. 10.1016/j.biocon.2015.01.012

[ece370306-bib-0071] European Commission . (2021). Natura 2000. European Commission. https://ec.europa.eu/environment/nature/natura2000/

[ece370306-bib-0015] Fabian, Y. , Sandau, N. , Bruggisser, O. T. , Aebi, A. , Kehrli, P. , Rohr, R. P. , Naisbit, R. E. , & Bersier, L.‐F. (2013). The importance of landscape and spatial structure for hymenopteran‐based food webs in an agro‐ecosystem. Journal of Animal Ecology, 82(6), 1203–1214. 10.1111/1365-2656.12103 23863136

[ece370306-bib-0016] Forister, M. L. , Pelton, E. M. , & Black, S. H. (2019). Declines in insect abundance and diversity: We know enough to act now. Conservation Science and Practice, 1, e80. 10.1111/csp2.80

[ece370306-bib-0017] Ganuza, C. , Redlich, S. , Uhler, J. , Tobisch, C. , Rojas‐Botero, S. , Peters, M. K. , Zhang, J. , Benjamin, C. S. , Englmeier, J. , Ewald, J. , Fricke, U. , Haensel, M. , Kollmann, J. , Riebl, R. , Uphus, L. , Müller, J. , & Steffan‐Dewenter, I. (2022). Interactive effects of climate and land use on pollinator diversity differ among taxa and scales. Science Advances, 8, eabm9359. 10.1126/sciadv.abm9359 35544641 PMC9075793

[ece370306-bib-0018] Gathmann, A. , Greiler, H.‐J. , & Tscharntke, T. (1994). Trap‐nesting bees and wasps colonizing set‐aside fields: Succession and body size, management by cutting and sowing. Oecologia, 98, 8–14. 10.1007/BF00326084 28312790

[ece370306-bib-0019] Guo, F. , Lenoir, J. , & Bonebrake, T. C. (2018). Land‐use change interacts with climate to determine elevational species redistribution. Nature Communications, 9, 1315. 10.1038/s41467-018-03786-9 PMC5883048 29615626

[ece370306-bib-0020] Habel, J. C. , Samways, M. J. , & Schmitt, T. (2019). Mitigating the precipitous decline of terrestrial European insects: Requirements for a new strategy. Biodiversity and Conservation, 28, 1343–1360. 10.1007/s10531-019-01741-8

[ece370306-bib-0021] Hall, M. , Nimmo, D. , Cunningham, S. , Walker, K. , & Bennett, A. (2019). The response of wild bees to tree cover and rural land use is mediated by species' traits. Biological Conservation, 231, 1–12. 10.1016/j.biocon.2018.12.032

[ece370306-bib-0022] Hallmann, C. A. , Ssymank, A. , Sorg, M. , de Kroon, H. , & Jongejans, E. (2021). Insect biomass decline scaled to species diversity: General patterns derived from a hoverfly community. Proceedings of the National Academy of Sciences of the United States of America, 118, e2002554117. 10.1073/pnas.2002554117 33431568 PMC7812780

[ece370306-bib-0023] Hanspach, J. , Hartel, T. , Horcea‐Milcu, I. , Mikulcak, F. , Dorresteijn, I. , Loos, J. , von Wehrden, H. , Kuemmerle, T. , Abson, D. , Kovács‐Hostyánszki, A. , Báldi, A. , & Fischer, J. (2014). A holistic approach to studying social‐ecological systems and its application to southern Transylvania. Ecology and Society, 19, 32. 10.5751/ES-06915-190432

[ece370306-bib-0024] Hartig, F. (2020). DHARMa – an R package for residual diagnostics of GLMMs. Theoretical ecology . https://theoreticalecology.wordpress.com/2016/08/28/dharma‐an‐r‐package‐for‐residual‐diagnostics‐of‐glmms/

[ece370306-bib-0025] Hoekstra, J. M. , Boucher, T. M. , Ricketts, T. H. , & Roberts, C. (2005). Confronting a biome crisis: Global disparities of habitat loss and protection. Ecology Letters, 8, 23–29. 10.1111/j.1461-0248.2004.00686.x

[ece370306-bib-0026] Hoffmann, U. S. , Jauker, F. , Diehl, E. , Mader, V. , Fiedler, D. , Wolters, V. , & Diekötter, T. (2020). The suitability of sown wildflower strips as hunting grounds for spider‐hunting wasps of the genus *Trypoxylon* depends on landscape context. Journal of Insect Conservation, 24, 125–131. 10.1007/s10841-019-00190-6

[ece370306-bib-0027] Holzschuh, A. , Steffan‐Dewenter, I. , & Tscharntke, T. (2009). Grass strip corridors in agricultural landscapes enhance nest‐site colonization by solitary wasps. Ecological Applications, 19, 123–132. 10.1890/08-0384.1 19323177

[ece370306-bib-0028] Holzschuh, A. , Steffan‐Dewenter, I. , & Tscharntke, T. (2010). How do landscape composition and configuration, organic farming and fallow strips affect the diversity of bees, wasps and their parasitoids? The Journal of Animal Ecology, 79(2), 491–500. 10.1111/j.1365-2656.2009.01642.x 20015213

[ece370306-bib-0030] Humbert, J.‐Y. , Pellet, J. , Buri, P. , & Arlettaz, R. (2012). Does delaying the first mowing date benefit biodiversity in meadowland? Environmental Evidence, 1, 1:9. 10.1186/2047-2382-1-9

[ece370306-bib-0031] Husson, F. , Josse, J. , Lê, S. , & Mazet, J. (2014). FactoMineR: Multivariate exploratory data analysis and data mining with R . R Package Version 1, 102–123.

[ece370306-bib-0070] Kassambara, A. , & Mundt, F. (2020). factoextra: Extract and visualize the results of multivariate data analyses. R package version 1.0.7. https://CRAN.R‐project.org/package=factoextra

[ece370306-bib-0032] Kovács‐Hostyánszki, A. , Földesi, R. , Mózes, E. , Szirák, Á. , Fischer, J. , Hanspach, J. , & Báldi, A. (2016). Conservation of pollinators in traditional agricultural landscapes – new challenges in Transylvania (Romania) posed by EU accession and recommendations for future research. PLoS One, 11, e0151650. 10.1371/journal.pone.0151650 27285118 PMC4902286

[ece370306-bib-0033] Kruess, A. , & Tscharntke, T. (2002). Grazing intensity and the diversity of grasshoppers, butterflies, and trap‐nesting bees and wasps. Conservation Biology, 16, 1570–1580. 10.1046/j.1523-1739.2002.01334.x

[ece370306-bib-0037] Lajos, K. A. (2022). Tájmetriai mutatók alkalmazása tájszerkezeti hatások felderítésére. PhD thesis, Magyar Agrár‐ és Élettudományi Egyetem. 10.54598/001900

[ece370306-bib-0034] Lajos, K. , Csaszar, O. , Sarospataki, M. , Samu, F. , & Toth, F. (2020). Linear woody landscape elements may help to mitigate leaf surface loss caused by the cereal leaf beetle. Landscape Ecology, 35, 2225–2238. 10.1007/s10980-020-01097-3

[ece370306-bib-0035] Lajos, K. , Demeter, I. , Mák, R. , Adalbert, B. , & Sárospataki, M. (2021). Preliminary assessment of cavity‐nesting hymenopterans in a low‐intensity agricultural landscape in Transylvania. Ecology and Evolution, 11, 1–12. 10.1002/ece3.7956 PMC8427617 34522349

[ece370306-bib-0036] Lajos, K. , Samu, F. , Bihaly, Á. D. , Fülöp, D. , & Sárospataki, M. (2021). Landscape structure affects the sunflower visiting frequency of insect pollinators. Scientific Reports, 11(1), 8147. 10.1038/s41598-021-87650-9 33854143 PMC8046751

[ece370306-bib-0038] Lanner, J. , Kratschmer, S. , Petrović, B. , Gaulhofer, F. , Meimberg, H. , & Pachinger, B. (2020). City dwelling wild bees: How communal gardens promote species richness. Urban Ecosystems, 23, 271–288. 10.1007/s11252-019-00902-5

[ece370306-bib-0039] Liu, J. , Wilson, M. , Hu, G. , Liu, J. , Wu, J. , & Yu, M. (2018). How does habitat fragmentation affect the biodiversity and ecosystem functioning relationship? Landscape Ecology, 33, 341–352. 10.1007/s10980-018-0620-5

[ece370306-bib-0040] McGarigal, K. , Cushman, S. , Neel, M. , & Ene, E. (2002). FRAGSTATS: Spatial pattern analysis program for categorical maps. University of Massachusetts. www.umass.edu/landeco/research/fragstats/fragstats.html

[ece370306-bib-0043] Nicholls, C. , & Altieri, M. (2012). Plant biodiversity enhances bees and other insect pollinators in agroecosystems. A review. Agronomy for Sustainable Development, 33, 257–274. 10.1007/s13593-012-0092-y

[ece370306-bib-0044] Nieto, A. , Roberts, S. , Kemp, J. , Rasmont, P. , Kuhlmann, M. , García Criado, M. , Biesmeijer, J. , Bogusch, P. , Dathe, H. , De la Rua, P. , De Meulemeester, T. , Dehon, M. , Alexandre, D. , Ortiz‐Sanchez, F. , Lhomme, P. , Pauly, A. , Potts, S. , Praz, C. , Quaranta, M. , & Michez, D. (2014). European Red List of bees. Publication Office of the European Union. 10.2779/77003

[ece370306-bib-0045] Oksanen, J. , Blanchet, F. G. , Friendly, M. , Kindt, R. , Legendre, P. , McGlinn, D. , Minchin, P. R. , O'Hara, R. B. , Simpson, G. L. , Solymos, P. , Stevens, M. H. H. , Szoecs, E. , & Wagner, H. (2019). vegan: Community ecology package .

[ece370306-bib-0046] Pan, Y. , Birdsey, R. , Phillips, O. , & Jackson, R. (2013). The structure, distribution, and biomass of the world's forests. Annual Review of Ecology, Evolution, and Systematics, 44, 593–622. 10.1146/annurev-ecolsys-110512-135914

[ece370306-bib-0047] Papanikolaou, A. D. , Kühn, I. , Frenzel, M. , & Schweiger, O. (2017). Semi‐natural habitats mitigate the effects of temperature rise on wild bees. Journal of Applied Ecology, 54, 527–536. 10.1111/1365-2664.12763

[ece370306-bib-0048] Paradis, E. , & Schliep, K. (2019). ape 5.0: An environment for modern phylogenetics and evolutionary analyses in R. Bioinformatics, 35, 526–528. 10.1093/bioinformatics/bty633 30016406

[ece370306-bib-0049] Pinheiro, J. , Bates, D. , DebRoy, S. S. , & Sarkar, D. (2013). Nlme: Linear and nonlinear mixed effects models . R package version 31‐110, 3, 1–113.

[ece370306-bib-0050] Potts, S. G. , Biesmeijer, J. C. , Kremen, C. , Neumann, P. , Schweiger, O. , & Kunin, W. E. (2010). Global pollinator declines: Trends, impacts and drivers. Trends in Ecology & Evolution, 25, 345–353. 10.1016/j.tree.2010.01.007 20188434

[ece370306-bib-0051] Potts, S. G. , Imperatriz‐Fonseca, V. , Ngo, H. T. , Aizen, M. A. , Biesmeijer, J. C. , Breeze, T. D. , Dicks, L. V. , Garibaldi, L. A. , Hill, R. , Settele, J. , & Vanbergen, A. J. (2016). Safeguarding pollinators and their values to human well‐being. Nature, 540, 220–229. 10.1038/nature20588 27894123

[ece370306-bib-0072] QGIS Development Team . (2009). QGIS Geographic Information System. Open Source Geospatial Foundation. http://qgis.osgeo.org

[ece370306-bib-0052] R Core Team . (2020). European Environment Agency [WWW document] . https://www.eea.europa.eu/data‐and‐maps/indicators/oxygen‐consuming‐substances‐in‐rivers/r‐development‐core‐team‐2006

[ece370306-bib-0053] Rader, R. , Reilly, J. , Bartomeus, I. , & Winfree, R. (2013). Native bees buffer the negative impact of climate warming on honey bee pollination of watermelon crops. Global Change Biology, 19, 3103–3110. 10.1111/gcb.12264 23704044

[ece370306-bib-0056] Schüepp, C. , Herrmann, J. D. , Herzog, F. , & Schmidt‐Entling, M. H. (2011). Differential effects of habitat isolation and landscape composition on wasps, bees, and their enemies. Oecologia, 165, 713–721. 10.1007/s00442-010-1746-6 20730546

[ece370306-bib-0057] Squires, V. , Dengler, J. , Feng, H. , & Hua, L. (2018). Grasslands of the world: Diversity, management and conservation . 10.1201/9781498796262

[ece370306-bib-0058] Stangler, E. S. , Hanson, P. E. , & Steffan‐Dewenter, I. (2015). Interactive effects of habitat fragmentation and microclimate on trap‐nesting hymenoptera and their trophic interactions in small secondary rainforest remnants. Biodiversity and Conservation, 24, 563–577. 10.1007/s10531-014-0836-x

[ece370306-bib-0059] Steffan‐Dewenter, I. (2002). Landscape context affects trap‐nesting bees, wasps, and their natural enemies. Ecological Entomology, 27, 631–637. 10.1046/j.1365-2311.2002.00437.x

[ece370306-bib-0061] Sumner, S. , Law, G. , & Cini, A. (2018). Why we love bees and hate wasps. Ecological Entomology, 43, 836–845. 10.1111/een.12676

[ece370306-bib-0055] Sánchez‐Bayo, F. , & Wyckhuys, K. A. G. (2019). Worldwide decline of the entomofauna: A review of its drivers. Biological Conservation, 232, 8–27. 10.1016/j.biocon.2019.01.020

[ece370306-bib-0063] Tilman, D. , Fargione, J. , Wolff, B. , D'Antonio, C. , Dobson, A. , Howarth, R. , Schindler, D. , Schlesinger, W. H. , Simberloff, D. , & Swackhamer, D. (2001). Forecasting agriculturally driven global environmental change. Science, 292, 281–284. 10.1126/science.1057544 11303102

[ece370306-bib-0064] Tormos, J. , Asís, J. D. , Gayubo, S. F. , Calvo, J. , & Martín, M. A. (2005). Ecology of crambonid wasps found in trap nests from Spain (Hymenoptera: Sphecifomes). Florida Entomologist, 88, 278–284. 10.1653/0015-4040(2005)088[0278:EOCWFI]2.0.CO;2

[ece370306-bib-0065] Tscharntke, T. , Gathmann, A. , & Steffan‐Dewenter, I. (1998). Bioindication using trap‐nesting bees and wasps and their natural enemies: Community structure and interactions. Journal of Applied Ecology, 35, 708–719.

[ece370306-bib-0066] Tscharntke, T. , Klein, A. , Kruess, A. , Steffan‐Dewenter, I. , & Thies, C. (2005). Landscape perspectives on agricultural intensification and biodiversity—Ecosystem service management. Ecology Letters, 8, 857–874. 10.1111/j.1461-0248.2005.00782.x

[ece370306-bib-0068] Wehn, S. , Burton, R. , Riley, M. , Johansen, L. , Hovstad, K. A. , & Rønningen, K. (2018). Adaptive biodiversity management of semi‐natural hay meadows: The case of West‐Norway. Land Use Policy, 72, 259–269. 10.1016/j.landusepol.2017.12.063

[ece370306-bib-0073] Wickham, H. (2016). ggplot2: Elegant graphics for data analysis (2nd ed.). Springer International Publishing. 10.1007/978-3-319-24277-4

[ece370306-bib-0069] Wilson, J. , Peet, R. , Dengler, J. , & Pärtel, M. (2012). Plant species richness: The world records. Journal of Vegetation Science, 23, 796–802. 10.2307/23251355

