# Supplementary material for: The role of wild bees and cavity‐nesting wasps as ecological indicators of the last traditionally managed meadows in Eastern Europe
Source: Ecol Evol. 2024 Oct 21;14(10):e70306. doi: 10.1002/ece3.70306 (PMC11493474; doi:10.1002/ece3.70306)
Supplement: Supplementary file 1 — Figures S1–S2 [file ECE3-14-e70306-s001.docx]

**Supplementary material**


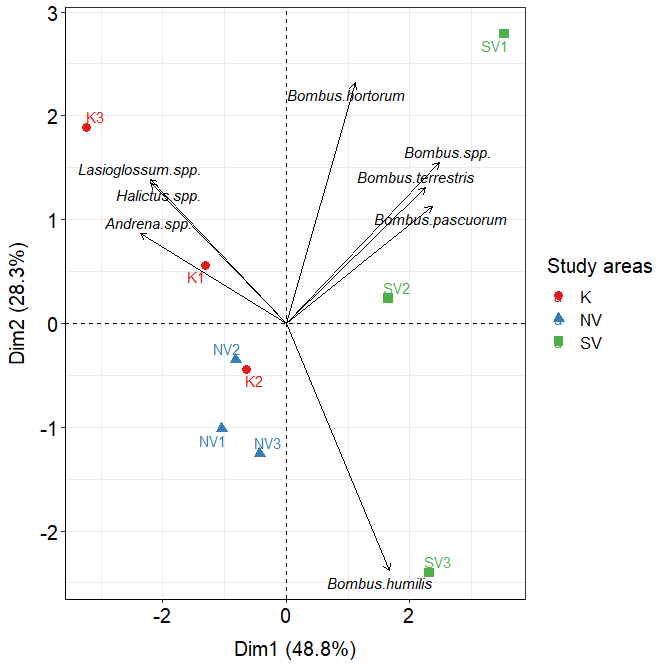

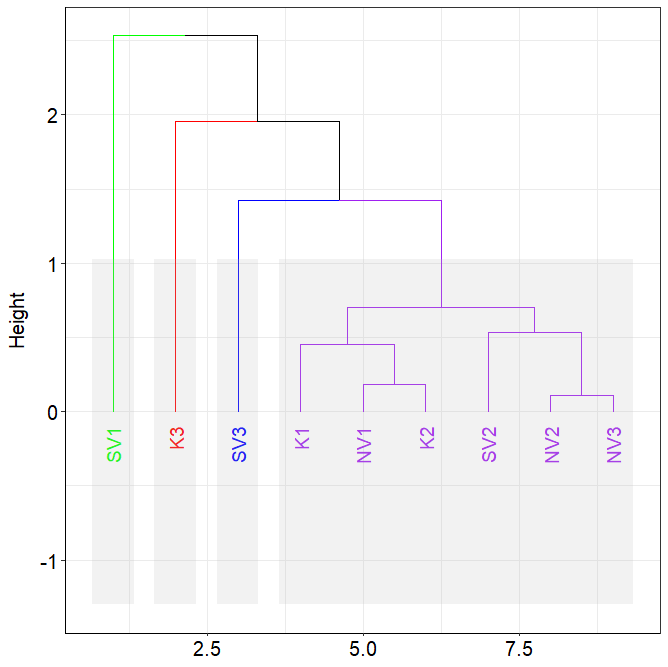

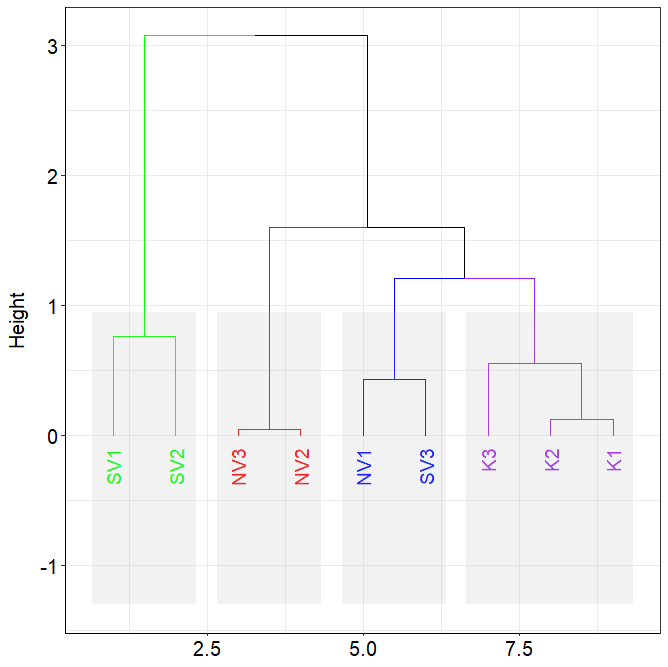

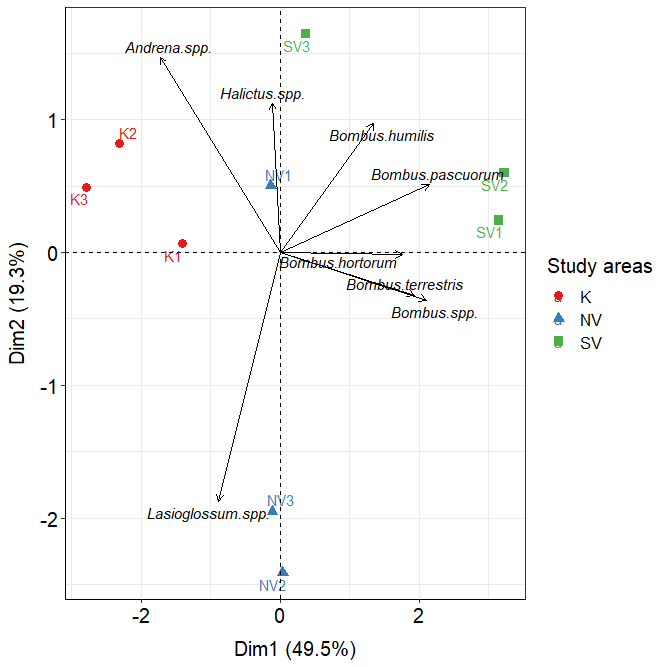


**(d)**

**(c)**

**(b)**

**(a)**

**Figure S1** Affinities of the most abundant wild bee taxa towards the study sites and areas in 2018 **(a, b)** and 2019 **(c, d)**. The affinities were determined using principal component analyses (PCAs) and dendrograms. The colors in the dendrograms do not correspond with the colors of the study areas in the figures depicting the PCAs.

**(a)**

**(b)**


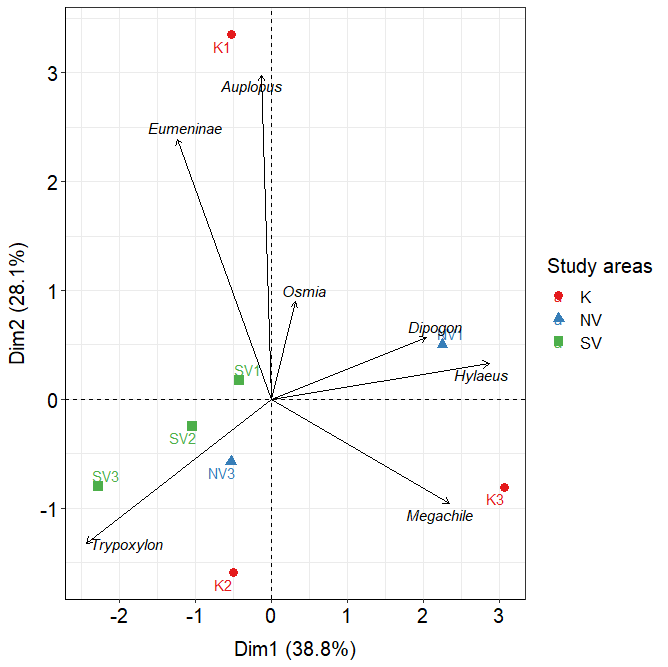

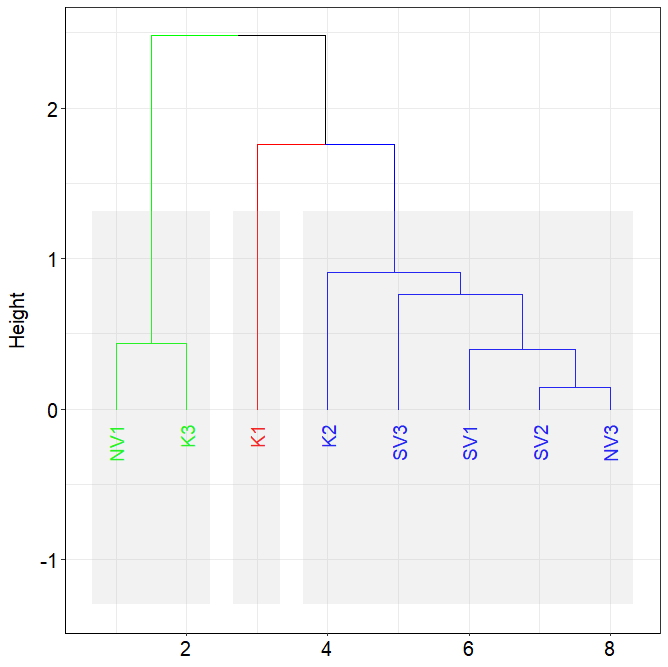


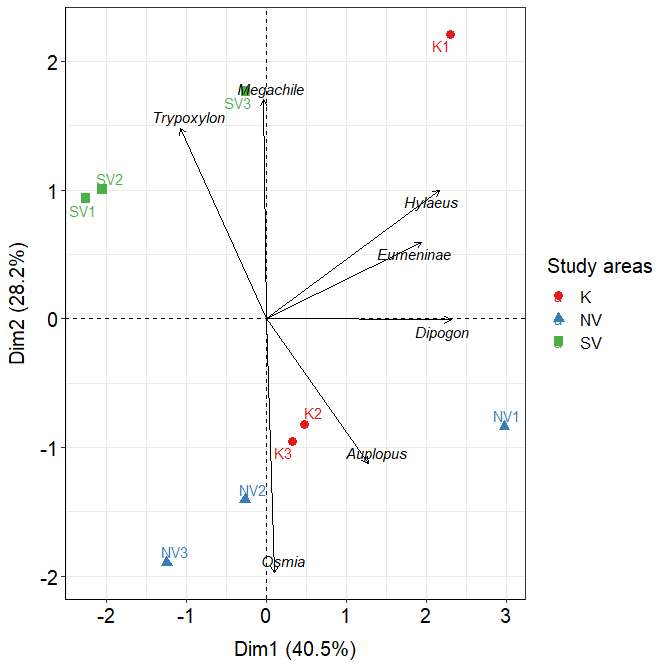

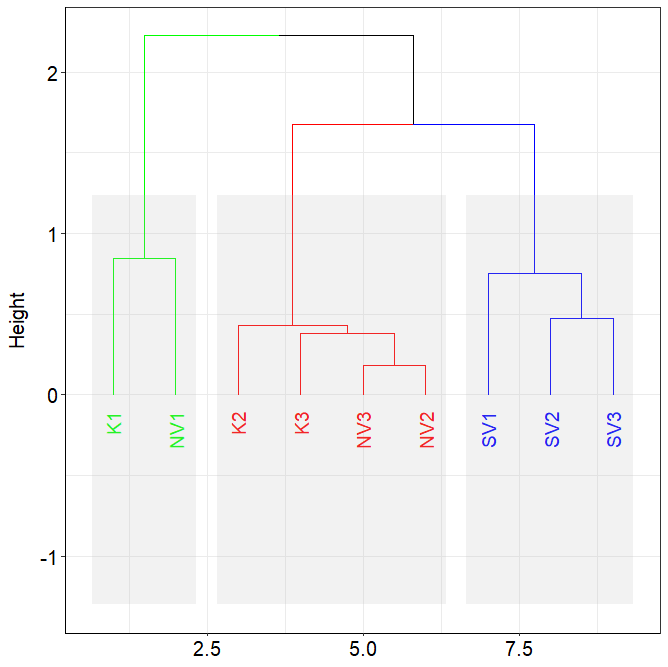


**(d)**

**(c)**

**Figure S2** Affinities of the seven different Hymenopteran nest types towards the study sites and areas in 2018 **(a, b)** and 2019 **(c, d)**. The affinities were determined using principal component analyses (PCAs) and dendrograms. The colors in the dendrograms do not correspond with the colors of the study areas in the figures depicting the PCAs.
